# Effects of Al^3+^ and La^3+^ Trivalent Metal Ions on Tomato Fruit Proteomes

**DOI:** 10.3390/proteomes5010007

**Published:** 2017-02-11

**Authors:** Sasikiran Sangireddy, Ikenna Okekeogbu, Zhujia Ye, Suping Zhou, Kevin J. Howe, Tara Fish, Theodore W. Thannhauser

**Affiliations:** 1 Department of Agricultural and Environmental Sciences, College of Agriculture, Human and Natural Sciences, Tennessee State University, 3500 John Merritt Blvd, Nashville, TN 37209, USA; sangisasi@gmail.com (S.S.); iyk_oc@yahoo.com (I.O.); zye@my.tnstate.edu (Z.Y.); 2 R.W. Holley Center for Agriculture and Health, USDA-ARS, Cornell University, Ithaca, NY 14853, USA; kjh46@cornell.edu (K.J.H.); tlf26@cornell.edu (T.F.)

**Keywords:** secondary metabolites, stress proteins, cell wall, photosynthesis, iTRAQ, functional pathways

## Abstract

The tomato (*Solanum lycopersicum*) ripening process from mature green (MG) to turning and then to red stages is accompanied by the occurrences of physiological and biochemical reactions, which ultimately result in the formation of the flavor, color and texture of ripe fruits. The two trivalent metal ions Al^3+^ and La^3+^ are known to induce different levels of phytotoxicity in suppressing root growth. This paper aims to understand the impacts of these two metal ions on tomato fruit proteomes. Tomato ‘Micro-Tom’ plants were grown in a hydroponic culture system supplemented with 50 μM aluminum sulfate (Al_2_ (SO4)_3_.18H_2_O) for Al^3+^ or La_2_(SO_4_)_3_ for La^3+^. Quantitative proteomics analysis, using isobaric tags for relative and absolute quantitation, were performed for fruits at MG, turning and red stages. Results show that in MG tomatoes, proteins involved in protein biosynthesis, photosynthesis and primary carbohydrate metabolisms were at a significantly lower level in Al-treated compared to La-treated plants. For the turning and red tomatoes, only a few proteins of significant differences between the two metal treatments were identified. Results from this study indicate that compared to La^3+^, Al^3+^ had a greater influence on the basic biological activities in green tomatoes, but such an impact became indistinguishable as tomatoes matured into the late ripening stages.

## 1. Introduction

Tomato (*Solanum lycopersicum*) is one of the most important fleshy vegetable crops in the world. According to the United States Department of Agriculture (USDA), based on the external color changes, the ripeness stages for red-fleshed tomatoes are divided into (1) mature green (MG), (2) breaker, (3) turning, (4) pink, (5) light red and (6) red [1]. Tomato fruits are predominantly composed of parenchyma cells enclosed by an unlignified layer of cellulose microfibrils suspended in a matrix of glycoproteins, water, pectic and hemicellulose polysaccharides [2]. Tomato ripening involves a variety of biochemical reactions in the flesh tissues, for example: the accumulation of lycopene leads to tomato turning red; an elevated content of citrate and malate makes the pulp acidic; the biosynthesis of a plentiful assortment of secondary metabolites gives the pleasant flavors and aroma; and the alteration of cell wall structures results in changes in fruit texture [3,4]. Underlying the fruit ripening process is a coordinated expression of sets of genes regulated by ripening-related transcription factors (TFs) and/or fruit-related microRNAs [5]. Tomato growth and production require optimum soil and air conditions. Plants growing on saline and/or drought-stricken soil and under chilling or hot temperatures produce low yield and poor fruit quality [6,7]. 

Excessive release of trivalent Al^3+^ ions is a common abiotic stress on acidic soil, which constitutes 30%–40% of agricultural land, and it is a major factor limiting crop production globally [8]. Tomato is sensitive to Al toxicity [9,10,11] at both the vegetative and reproductive stages. Previous studies have shown that Al treatments induced global changes in proteome expression in roots, leaves, fruits and seeds in tomato [12,13,14]. These molecular responses are mobilized to deal with Al^3+^ ion toxicity, as well as the Al-induced interferences in plant mineral nutrition [13,15]. 

Trivalent metal ions include aluminum (III), lanthanide (III), iron (III) and chromium (III). In earlier studies of Al toxicity, the trivalent lanthanide ions (La^3+^) was added in the control treatments to balance the ion strength in the basic nutrient solution [16]. However, La^3+^ ions also interact with plant cells in a similar wat as Al^3+^; for instance, both La^3+^ and Al^3+^ ions can bind to the negative groups of the phospholipids of the cell membrane [17,18,19]. In wheat (*Triticum aestivum*) and maize (*Zea mays*), the root cap tissues were damaged under either Al^3+^ or La^3+^ treatments, and the resulting ion toxicity symptoms were ameliorated by the addition of cations (such as Ca^2+^) [18,20]. La^3+^ was once suggested as an analog of Al^3+^ to be used as a biological tracer for studying Al toxicity [20,21]. On the other hand, there exists a great difference between these two ion species. Al^3+^ has the smallest ionic radius (0.54 Å) in contrast to the largest ionic radius of La^3+^ (1.03 Å) among the trivalent ions, and the former species has higher binding energy strength with disaccharide chains [22]. These physical properties may affect their capacity of displacing essential ions on the cell membrane, such as Ca^2+^ (0.99 Å), which is larger than Al^3+^, but slightly smaller than La^3+^. Taking into consideration the essential role of Ca^2+^ in maintaining cell homeostasis, it is conceivable that Al^3+^ is more harmful to plants than La^3+^. Indeed, studies have shown that plants are more sensitive to Al than La [23]. These physiological studies have revealed similarity, as well as differences in phytotoxicity between these two trivalent ions, but molecular studies are needed to understand and validate these differential impacts on biological systems. 

The proteome is defined as the entire sets of proteins expressed in a given tissue at specific time points. Proteomics analysis has proven to be a very powerful tool in revealing the relative abundance of proteins or the identification of individual proteins related to specific cell functions in plants [24,25,26]. More importantly, our previous studies have shown that Al treatments affected proteome composition, not only in roots, but also in the aboveground tissues, including leaves, cotyledons, seeds and germinating radicles derived from the Al-treated seeds [12,25,26]. 

In this study, we compared the proteome changes as tomatoes ripened from MG, to turning and then to red stages and also to identify the impacts of Al vs. La treatments on these tomato proteins. Some of the protein changes concur with the physiological properties of tomato ripening stages. For instance, proteins in the synthesis pathways of secondary metabolites were enriched in turning and red tomatoes, but not in MG tomatoes. In contrast, the Al-repressed proteins in the photosynthesis pathway were identified in MG tomatoes, but not in the other two ripening stages. Findings from this study will be very useful to establish the correlation between proteome expression and metabolite composition in tomatoes at different ripening stages. 

## 2. Materials and Methods 

### 2.1. Plant Material and Treatment

The miniature tomato ‘Micro-Tom’ bearing red tomatoes was used in this study. Seed stocks were provided by Lázaro E. P. Peres, University of São Paulo, Brazil, and they were propagated in a greenhouse at Tennessee State University in Nashville, TN, USA. For this experiment, 300 seeds were surface-sterilized by soaking in 100 mL of 30% (*v*/*v*) commercial bleach and washed with distilled water 3 times. Seeds were kept in Magenta box with water and placed on a rotary shaker for 2 days. Germinating seeds were transferred to Oasis root cubes (Smithers-Oasis, Kent, OH, USA). At the two-leaf stage, plants were placed in net pots with the support of hydroton clay balls and transplanted into hydroponic tanks filled with 30 L of a modified Magnavaca’s solution (pH, 4.5) [27]. There were eight tanks each growing 18 plants. 

**Figure 1:**
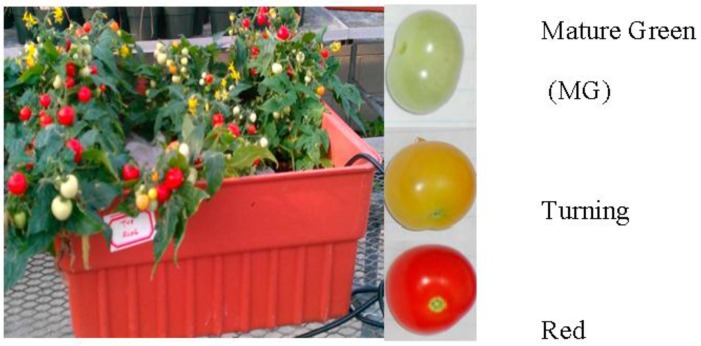
Tomato ‘Micro-Tom’ plants growing in the hydroponic tank and tomato fruits harvested for analysis (mature green, turning and red indicate the ripening stages of the tomatoes).

Previously, aluminum sulfate octadecahydrate (Al_2_(SO_4_)_3_·18H_2_O) and lanthanum (׀׀׀) sulfate hydrate (La_2_(SO_4_)_3_) were used in plant treatment experiments [20,28]. In this study of tomatoes, Al was added to a final concentration of 50 μM Al_2_(SO_4_)_3_·18H_2_O (Fisher Scientific, Pittsburgh, PA, USA) in the Al-treated group and La of 50 μM La_2_(SO_4_)_3_ (Strem chemicals, MA, USA) in the La-treated group. These treatments were initiated when two or more tomatoes on the first fruit cluster turned red. The pH of the solution was monitored daily using pH indicator strips (Fisher Scientific). The solution was refreshed every three days. After nine days of treatments, tomato fruits in the MG, turning and red stages (Figure 1) were harvested. Two fruits were taken from each plant; samples from one tank were pooled to form one biological replicate. Four biological replicates each for Al-treated and La-treated plants were harvested. Samples were frozen in liquid nitrogen immediately after collection and stored at −80 °C. 

### 2.2. Proteomic Analysis 

#### 2.2.1. Protein Extraction

To extract protein, frozen tomatoes were ground into a fine powder under N_2_. Total protein was extracted using the Plant Total Protein Extraction Kit (Sigma, St. Louis, MO, USA), following the manufacturer’s instruction with modifications. Briefly, 5 g aliquots of the tissue powder were transferred into 50-mL centrifuge tubes to which 50 mL of the extraction solution (10% trichloroacetic acid (TCA)) in acetone was added. Samples were vortexed, incubated overnight at −20 °C and centrifuged at 10,000 rpm for ten minutes at 4 °C. Protein pellets were washed 3 times with pre-chilled acetone to remove residual TCA and dried briefly. Proteins were solubilized in a solution of 6 M urea/500 mM triethylammonium bicarbonate (TEAB) and then diluted (1:3, *v*/*v*) with TEAB buffer to reduce urea to a final concentration of 2 M. The protein concentration was determined by Bradford’s method using bovine serum albumin (BSA) as the standard (Bio-Rad, Hercules, CA, USA). 

#### 2.2.2. iTRAQ Labeling Procedure

An aliquot of 100 µg protein per sample was used for iTRAQ labeling following the instruction in the 8-plex iTRAQ^®^ labeling kit (AB SCIEX, Foster City, CA, USA). Sodium dodecyl sulfate (SDS) was added to the samples to a final concentration of 0.1%. To reduce the disulfide bonds of proteins, tris (2-carboxyethyl) phosphine (TCEP) was added to a final concentration of 5 mM, and the samples were incubated at 32 °C for 1 h. Next, the cysteines were blocked by adding methyl methane thiosulfonate (MMTS) to a final concentration of 10 mM, and the samples were incubated at room temperature for 30 min. For protein digestion, 2 µg of sequence-grade modified trypsin enzyme (Promega, Madison, WI, USA) were added to each sample, and samples were incubated at 30 °C for 18 h. After digestion, the volume of each sample was reduced to 20 µL, by vacuum centrifugation. The four Al-treated samples were labeled with iTRAQ Reagents 113, 114, 117 and 118, while the four La-treated control samples were labeled with iTRAQ Reagents 115, 116, 119 and 121. The labeling reaction was allowed to proceed for 2 h at room temperature, protected from light. 

#### 2.2.3. Cleaning of the Peptides

After the labeling reaction, the 8 samples were pooled into one tube, mixed and evaporated by vacuum centrifugation. Excess TCEP, SDS and iTRAQ reagents were removed from the sample using an iCAT cation exchange cartridge (AB Sciex, Framingham, MA, USA). The dried sample was resuspended in a cation exchange load buffer (10 mM potassium phosphate in 25% acetonitrile, pH 3.0). The pH of the sample was adjusted to pH 2.5 using trifluoroacetic acid (TFA). The iCAT cartridge was cleaned with 1 mL of the cation exchange clean buffer (10 mM potassium phosphate in 25% acetonitrile/1M potassium chloride, pH 3.0). The cartridge was conditioned with 2 mL of the cation exchange load buffer. The sample mixture was slowly injected onto the iCAT cartridge. To wash the peptides, 2 mL of the cation exchange load buffer were injected through the cartridge. The labeled peptides were eluted from the cartridge and collected by injecting 600 uL of the cation exchange elution buffer (10 mM potassium phosphate in 25% acetonitrile/350 mM potassium chloride, pH 3.0) through the cartridge. The elution was dried to completion by vacuum centrifugation. 

The sample was resuspended in 0.1% TFA for a solid phase extraction (SPE) step to remove salts. The pH of the sample mixture solution was brought to 3 with TFA. A SepPak C18, 1 mL, vacuum cartridge (Waters, Milford, MA, USA) was cleaned using acetonitrile. Then, the cartridge was conditioned with 5 mL of 0.1% TFA. The sample mixture was slowly loaded through the cartridge using a needle valve to control the vacuum. The cartridge was washed with 5 mL of 2% acetonitrile/0.1% TFA. The peptides were then eluted with 600 uL of 50% acetonitrile/0.1% TFA. The eluate was dried to completion and submitted for analysis of the iTRAQ-labeled peptides. 

#### 2.2.4. High pH First Dimension UPLC Separation

A Waters Acquity UPLC (Milford, MA, USA) coupled with a Dionex Probot (Sunnyvale, CA, USA) robotic fraction collector was used to separate the peptides. A Waters Acquity UPLC BEH C18 1.7 µm, 2.1 mm × 100 mm column was used. Mobile Phase A consisted of 20 mM ammonium formate dissolved in water (pH 10), and Mobile Phase B consisted of 90:10 acetonitrile: water (*v*/*v*) containing 20 mM ammonium formate (pH 10). For the separation, a segmented gradient was used employing a flow rate of 300 µL/min. The initial conditions were 0% B; Segment 1 was 0% ➔ 5% B over 0.5 min; Segment 2 was 5% ➔ 35% B (linear) over 8 min; and Segment 3 was 35% ➔ 95% B over 1 min. The column was then returned to initial conditions and re-equilibrated over 4 min. One hundred micrograms of total labeled protein digest were injected and separated into 48 fractions in a 96-well plate. The fraction collector was triggered via contact closure after the injection and was delayed for 30 s before commencing collection. A total of 48 fractions were collected, with one fraction being collected every 14 s. Fractions were then concatenated as follows: 1st Dimension Fractions, 1, 2, 3 and 4 were mixed to produce second dimension Sample 1; Fractions 5 and 25 were mixed to make Sample 2; Fractions 6 and 26 were mixed to make Sample 3; and so forth, to produce an additional 20 fractionally-concatenated second dimension samples. The 22nd sample was produced by pooling Fractions 45–48.

#### 2.2.5. Low pH Second Dimension RP Separation

Dried samples were reconstituted with 24 μL of 3% acetonitrile mixed with 0.1% TFA. A nanoACQUITY system (Waters), equipped with a Symmetry C18 5 µm, 20 mm × 180 µm trapping column and a UPLC BEH C18 1.7 µm, 15 cm × 75 µm analytical column (Waters), was used to perform the nano-LC separation of tryptic peptides. The samples, 3-μL partial loop injections, were transferred to the trapping column via a 0.1% solution of formic acid in water at a flow rate of 7 µL/min for three minutes. Mobile Phase A contained water with 0.1% formic acid, and B contained acetonitrile with 0.1% formic acid. The trapping column was subjected to a reverse flush to the analytical column after the desalting and concentration and was separated at a flow rate of 300 nL/min. The initial conditions were run using 2% B. A 3-segment gradient was used to elute the peptides: Segment 1, linear 2% ➔ 5% B over 2 min; Segment 2, linear 5% ➔ 10% B over 1 min; Segment 3, linear 10% ➔ 35% B over 2 h. The column was then washed for 5 min with 95% of Mobile Phase B before returning to initial conditions and a 20-min equilibration period. The column temperature was maintained at 35 °C and re-equilibrated at initial conditions for 20 min prior to the next injection. One hundred femtomoles per microliter [Glu1]-fibrinopeptide B in 25% acetonitrile with 0.1% formic acid were used as the lock mass compound and were delivered via the auxiliary pump of the LC system at a flow rate of 300 nL/min to the reference sprayer of the Nano Lock Spray source of the mass spectrometer. From the analytical column, the eluent was delivered to the analytical sprayer of the same source through a PicoTip emitter (New Objective, Woburn, MA, USA) with a 10-μm tip diameter.

#### 2.2.6. Mass Spectrometric Analysis

Tryptic peptides were subjected to mass spectrometric analysis, using a Synapt HDMS mass spectrometer (Waters). The Synapt was operated in Q-TOF V mode with a typical resolution of at least 10,000 full width at half maximum (FWHM). The TOF analyzer of the mass spectrometer was externally calibrated by fragmenting the doubly-protonated mono isotopic ion of [Glu]^1^-fibrinopeptide B and delivering it via the lock mass reference sprayer. Calibration was performed over the *m*/*z* range from 50–2000, and the collected data were then post-acquisition lock mass corrected. The reference sprayer was sampled for one second after every 100 s. Accurate mass LC-MS/MS data-dependent acquisition (DDA) data were obtained as follows: MS survey scans of one-second duration with an inter-scan delay of 0.02 s were acquired for the *m*/*z* range from 300–1500. The intensity of a single ion rising above a set 60 counts per second threshold triggered MS/MS fragmentation for the ion provided the ion met the charge state criteria. MS/MS data were obtained for up to four ions of charge 2^+^, 3^+^, or 4^+^ detected in the survey scans using charge state selection. MS/MS spectra were acquired for the *m*/*z* ranges from 50–1400 at a scan rate of 1 s with an inter-scan delay of 0.02 s. To improve the quality of MS/MS spectra, charge state-dependent collision energy ramps were optimized and employed. A real-time dynamic exclusion window of 35 s was applied to each precursor selected for fragmentation. The instrument returned to MS mode when the total ion current for an MS/MS acquisition exceeded 30,000 counts per second (cps) or after 2.5 s had elapsed.

#### 2.2.7. Data Processing

All raw data files were processed using ProteinLynx Global Server 2.4 into a .pkl peak list format compatible with Mascot (Matrix Science, Boston, MA,). The software was adjusted for mass accuracy by performing lock mass correction using the doubly-protonated, monoisotopic peak for [Glu]^1^-fibrinopeptide B at *m*/*z* 785.8426, utilizing 2 lock spray scans with a tolerance of 0.25 Dalton (Da). For noise reduction, both precursor and fragment functions were set to perform “adaptive” background subtraction. For deisotoping and centroiding, both functions instructed the software to perform deisotoping at the “medium” setting with automatic thresholds.

#### 2.2.8. Database Searching/iTRAQ Quantitation

The 22 packing list file (pkl) files from the 22 injections of each sample were combined using Mascot Daemon v. 2.3.2 to make a single query against an iTAG 2.3 tomato protein database (downloaded 16 September 2011; solgenomics.net/tomato/). The enzymatic cleavage specificity was set to trypsin with 1 missed cleavage allowed. Precursor tolerance was set at 25 ppm, while fragment tolerance was set to 0.1 Da. The instrument selected was electrospray ionization quadrupole time-of-flight mass spectrometry (ESI-QUAD-TOF). Individual iTRAQ quantitation methods were set up and used depending on the control/treatment/pool reporter labels used for each sample. Each quantitation method used “average” for the protein ratio type and required a minimum of 2 peptides for protein quantitation. For normalization, “average” was used. N-terminal and lysine modification with iTRAQ were set as fixed modifications, and tyrosine labeling was set as variable modification. Upon completion of the searches, results were exported after setting the ion score filter to 0.1, thereby exporting only results with an expectation value below 0.1 and requiring bold red in order to limit results to the highest scoring match to a particular query listed under the highest scoring protein containing that match. Functional pathways of the listed proteins were analyzed using the MapMan tools (Version 3.6.0RC1) [29,30]. Additionally, a literature search was also used to identify the functions of particular proteins relevant to tomato fruit ripening and Al and La stresses. 

### 2.3 Statistical Analysis 

Statistical analysis was done by using SAS (Version 9.3; SAS Institute, Cary, NC, USA). Raw expression ratios were log_2_ transformed and then fit to a normal distribution [25,31]. The log_2_ fold values were subjected to *t*-test using the general linear model (GLM) procedure followed by the false discovery rate corrections using SAS. Significant proteins were listed for those passing the two steps of statistical tests (*p* ≤ 0.05) and with the fold value either lower than 0.65 (<0.65) or higher than 1.51 (>1.51-fold), from Al-treated to La-treated groups. 

## 3. Results

### 3.1. The Al-Treatment-Induced Proteome Composition in Tomatoes 

**Figure 2:**
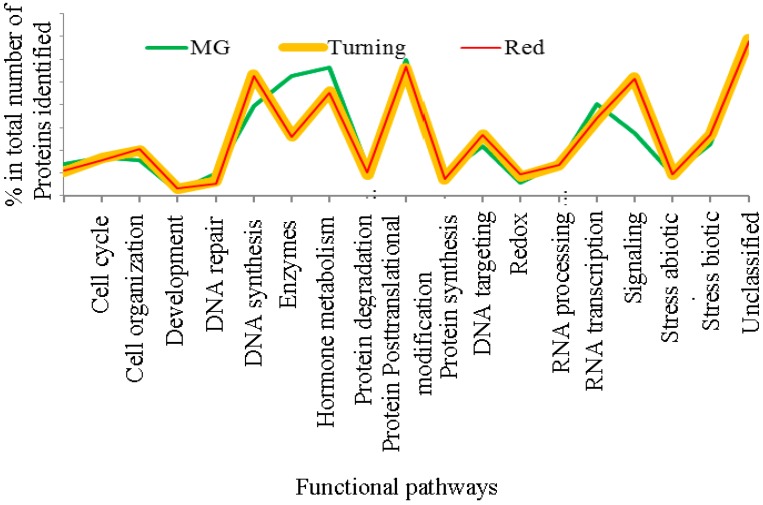
Distribution of functional pathways for proteins from mature green, turning and red stage tomatoes (Tomatoes at turning and red stages contain nearly the same protein distribution, and the mature green (MG) tomatoes show distinct distribution in enzymes, hormone metabolism, protein degradation and stress responses. The cellular pathways were constructed using the tools in MapMan [29,30].).

Using the criteria of two unique peptides per protein, 809 proteins were quantified for MG tomatoes and 600 proteins for both turning and red tomatoes each. When these proteins were analyzed for functional pathways in MapMan, it was found that protein expression in MG tomatoes differs from those from turning and red tomatoes, which display more similar protein expression patterns, as can be seen from Figure 2. 

### 3.2. Differentially-Expressed Proteins in Tomatoes under Al and La Treatments 

In MG tomatoes, compared to La^3+^, the treatments with Al^3+^ induced a more pronounced and consistent repression of proteins in the functional pathways for protein biosynthesis, photosynthesis, primary carbohydrate metabolic pathways and redox status regulation. When tomatoes reached the turning or red stages, very few proteins showed differential responses to these two metal ions (Figure S1). In Al-treated MG tomatoes, 17 significantly changed proteins were repressed (Table 1). These Al-repressed proteins include two cell wall proteins, sucrose synthase in sucrose metabolism/catabolism, phosphoglycerate kinase, transketolase, NADP-malic enzyme and aldehyde dehydrogenase in glycolysis and the TCA cycle and the cinnamyl-alcohol dehydrogenase in the secondary metabolic pathway. Six repressed proteins were classified in the RNA-protein synthesis and post-translational modification pathways. Four ribosomal proteins and the 26S proteasome regulatory subunit S2 1A were also repressed. The identification of the chloroplast photosystem II PsbB and RUBISCO in the MG tomatoes concurs with the physiological property that green tomatoes have some photosynthetic activity [32]. 

Saccharopine reductase is an enzyme involved in the metabolism of the amino acid lysine; this protein was more repressed in Al-treated MG fruits. The Al-induced proteins include two vicilin-like proteins at 1.5–2.0 fold (Log2, 0.6–0.99-fold), a protein for defective in meristem silencing at 1.5-fold (Log2, 0.6-fold) involved in chromatin remodeling, a FAR1 protein with a transposase function at 1.55-fold (Log2, 0.63-fold), annexin at 1.85-fold (Log2, 0.89-fold) involved in signaling, two stress proteins, including Cc-nbs-lrr at 1.57-fold (Log2,0.65-fold) and a blue copper protein at 2.39-fold (log2, 1.26-fold), and GDSL esterase/lipase at 1.92-fold (Log2, 0.94-fold) involved in lipid metabolism.

In the turning and red tomatoes, only a few proteins expressed significant differences between the Al-treated and La-treated groups. The lectin protein was consistently repressed, while the Kunitz trypsin inhibitor at 1.86-fold (Log2, 0.90-fold) was induced in Al-treated compared to La-treated groups. In turning tomatoes, acid beta-fructofuranosidase (Log2, −0.60-fold) catalyzing the hydrolysis of sucrose was repressed at −1.5-fold, whereas the ABSCISIC ACID STRESS RIPENING 1 protein (Log2, 0.60-fold) was induced at 1.51-fold, in the Al-treated as opposed to the La-treated group. Methionine sulfoxide reductase catalyzes the reduction of methionine sulfoxide in proteins back to methionine, and thus, it has been shown to play an important role in protecting cells from damage from reactive oxygen and nitrogen intermediates [33]. The protein was repressed in Al-treated red tomatoes at −1.53-fold (log2, −0.61-fold). 

### 3.3. Patterns of Proteome Changes during the Tomato Ripening Process Associated with Al and La Treatments 

**Figure 3:**
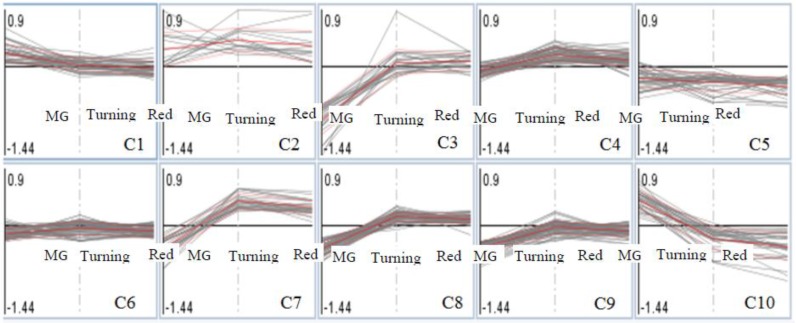
Cluster analysis of protein fold changes in Al-treated compared to La-treated tomatoes (the three points showing MG, turning, red stages of tomatoes; the analysis was conducted in MapMan [29,30]).

Proteins identified in MG, turning and red tomatoes were placed into 10 clusters (Figure 3), and those showing significant changes in any one of the three tomato ripening stages between Al-treated and La-treated groups (log2, ±0.6-fold, Al-treated/untreated; *p* < 0.05) are listed in Table 2. In Clusters 1 and 2, proteins were either induced by Al treatment or showed no difference with La-treated tomatoes. In this group, only one protein (Cc-nbs-lrr, resistance protein) passed the threshold for significant proteins in MG fruits. In Cluster 3, 15 proteins were significantly repressed in MG tomatoes, but not in turning and red tomatoes. Cluster 5 contains proteins that were repressed by the Al-treated compared to the La-treated group across the three ripening stages. Cluster 6 contains proteins showing no differences between Al-treated and La-treated groups. Cluster 7 contains proteins that were repressed by Al treatment in MG tomatoes, but induced in turning and red tomatoes. Cluster 9 contains proteins showing Al inducement in MG, but repressed in turning and red tomatoes compared to La treatments. Cluster 10 contains protein induced by Al-treatment in MG tomatoes, but repressed in turning and red tomatoes.

## 4. Discussion

This study identified changes in tomato proteomes as they ripened from MG to turning and then to the red fruits under Al or La treatment conditions. Green tomatoes contain intact chloroplasts and plastids [34]. Photosynthesis of these green tomatoes contributes a portion of carbohydrates to provide for fruit development. Studies have shown that disrupting the photosynthetic activities of these green tomatoes can lead to poor fruit quality [35,36,37], as well as early seed development [38]. Compared to the La treatment, MG tomatoes in the Al-treated group expressed a decline in proteins that are structural components of the photosystem II (PSII) and enzymes in nearly every step of the Calvin cycle, including RUBISCOs (Figure S2). These protein changes clearly show that Al^3+^ treatments induced a more harmful effect than La^3+^ in green tomatoes. Similar Al-induced leaf protein changes in the same biological pathway were reported previously [25]. These protein changes would explain the Al-induced decline in photosynthesis activity of tomato plants [10,11], and it is obvious that Al^3+^ is more damaging than La^3+^ to the respective biological process. 

As tomato ripens from MG to turning and red stages, chloroplasts are converted into chromoplasts, and this photosynthetic machinery is dismantled. According to the proteomics analysis, those photosynthetic proteins also diminished at these two latter ripening stages. Thus, the fruit proteome changes appear to concur with the physiological status of these tomatoes. Glycolysis and the citric acid cycle (TCA) provide substrates for the biosynthesis of organic acids, mainly malate and citrate in tomatoes [39,40]. The turning and red tomatoes are characterized by carotenoids’ accumulation in chromoplasts, as well as hexoses, glucose and fructose, organic acids, aromatic amino acids and secondary metabolites [41,42,43]. At the turning and red stages, the Al-treated tomatoes were more enriched with the N-carbamoylputrescine amidase, which catalyzes the final step for the conversion of putrescine. Accumulation of this polyamine can serve two roles: one is to provide some stress tolerance mechanisms to the cells, and the second mechanism is related to climacteric ethylene production to promote fruit ripening. Both endogenous and exogenous putrescine were shown to have a positive role in promoting fruit ripening in bananas (*Musa* AAA group) [44]. However, no such direct correlation has been established in tomato. Instead, several studies have shown that putrescine content in tomato pericarp is not related to normal ripening [45], and an increasing free polyamine level in ripe pericarp in the tomato cultivar Liberty may account for the reduction of climacteric ethylene production [46]. The ripening-specific accumulation of polyamines, spermidine and spermine allows these to act as antiapoptotic regulatory molecules that are able to revive metabolic memory in the tomato fruit [47]. Due to such significant roles of polyamines described above, the enrichment of proteins toward the production of putrescine in Al-treated tomatoes suggests that Al treatment may have some influences on the length of the tomato ripening process. 

Tomato fruit ripening is accompanied by seed development and maturation. In the MG stage tomatoes, the embryo has reached full size, but the seed coat is still light-colored with a tender structure. Beyond this point, the seed maturation stage starts with the characteristic dehydration process. In the turning stage tomatoes, Al treatment induced a higher abundance level of ABSCISIC ACID STRESS RIPENING1 protein 1 (ASR1), which is a low molecular weight plant-specific protein encoded by a salt-, drought- and ABA-regulated gene [48,49,50]. Additionally, the Al-treated red tomatoes were also more enriched with hydrophilic proteins, as well as stress proteins. Among these proteins, vicilin-like 7S globulins are a class of main seed storage proteins; they perform a plethora of functions in maintaining protein structural stability against oxidative cellular stresses during embryo development [51]. Previous studies also found that stress proteins were more enriched in tomato seeds derived from Al-treated tomato plants [25], as well as in the germinating radicles upon Al treatments [12]. Thus, we can conclude that tomatoes and seeds grown under Al-treated conditions contain an abundance of stress proteins, which may have a role in preparing the progeny of these plants for the same stress factors. 

## 5. Conclusions 

This study has established the influences of different trivalent cations (Al versus La) on tomato proteomes during fruit development and ripening. Results from the proteomics analysis clearly indicate that an excessive Al level has a much greater impact than La on protein abundance for enzymes in nearly all of the primary and secondary metabolic pathways in MG tomatoes. However, these influences became attenuated or diminished in more mature tomatoes as they ripened to the turning and red tomato stages. These protein changes in tomato fruits in response to Al treatments firmly confirmed the influences of Al toxicity on tomato fruit development and ripening. The study has identified a list of proteins and the candidate cellular functional pathways that responded to Al or/and La toxicity. These results clearly indicate that for fruit metabolome studies, it is imperative to take into consideration the impacts of environmental conditions on fruit physiological properties, including fruit photosynthesis, primary and secondary metabolites, flavor development and many others. 

## Figures and Tables

**Table 1:** Major functional pathways constructed using proteins identified in tomatoes ^a^.

Log2 Fold (Al/La) ^b^	FDR (*p*-Value)	Protein Accession Number ^c^	Protein Description
Mature green (MG) tomatoes			
−1.44	0.05	Solyc11g012320.1.1	Unknown protein
−1.36	0.02	Solyc10g075090.1.1	Non-specific lipid-transfer protein
−1.27	0.03	Solyc05g051260.2.1	Endo-1 4-beta-xylanase
−0.97	0.04	Solyc02g094470.2.1	Mitochondrial phosphate carrier protein
−0.93	0.01	Solyc01g104590.2.1	Ribosomal protein L3
−0.90	0.04	Solyc01g007500.2.1	Photosystem II CP47 chlorophyll apoprotein
−0.89	0.03	Solyc01g109970.2.1	DNA repair protein
−0.89	0.03	Solyc00g035010.1.1	C2H2 finger domain-containing protein
−0.89	0.03	Solyc11g069660.1.1	Nbs-lrr, resistance protein
−0.89	0.03	Solyc07g007760.2.1	Defensin protein
−0.88	0.03	Solyc12g009300.1.1	Sucrose synthase
−0.84	0.03	Solyc03g122310.2.1	Aldehyde dehydrogenase
−0.81	0.01	Solyc06g071720.1.1	60S ribosomal protein L27A
−0.80	0.02	Solyc02g090560.2.1	Calcium-transporting ATPase
−0.74	0.00	Solyc01g007330.2.1	Ribulose bisphosphate carboxylase large chain
−0.71	0.03	Solyc11g065240.1.1	Saccharopine dehydrogenase
−0.70	0.01	Solyc06g060340.2.1	Chloroplast photosystem II-associated protein
−0.70	0.04	Solyc07g053650.2.1	26S proteasome regulatory subunit
−0.69	0.00	Solyc11g062130.1.1	Mitochondrial ADP/ATP carrier proteins
−0.68	0.04	Solyc04g063290.2.1	30S ribosomal protein S5
−0.68	0.02	Solyc07g066610.2.1	Phosphoglycerate kinase
−0.66	0.00	Solyc06g009190.2.1	Pectinesterase
−0.66	0.04	Solyc01g107590.2.1	Cinnamyl alcohol dehydrogenase
−0.65	0.00	Solyc05g054580.2.1	60S acidic ribosomal protein P0
−0.63	0.01	Solyc12g044600.2.1	NADP-dependent malic enzyme, chloroplastic
−0.62	0.01	Solyc02g078570.2.1	Epoxide hydrolase 3
−0.61	0.01	Solyc10g018300.1.1	Transketolase 1
0.60	0.00	Solyc03g083170.2.1	Defective in meristem silencing 3 (chromatin remodeling)
0.60	0.00	Solyc09g082330.1.1	7S vicilin
0.61	0.05	Solyc01g060070.2.1	Pore protein homolog
0.63	0.02	Solyc06g065940.2.1	Protein FAR1-RELATED SEQUENCE 6
0.65	0.02	Solyc03g005650.1.1	Cc-nbs-lrr, resistance protein
0.72	0.00	Solyc07g063120.2.1	WD-40 repeat protein
0.89	0.00	Solyc04g073990.2.1	Annexin
0.94	0.03	Solyc01g079160.2.1	GDSL esterase/lipase
0.99	0.01	Solyc09g082350.1.1	Vicilin-like protein
1.26	0.04	Solyc05g054900.2.1	Blue copper protein
Turning tomatoes			
−0.61	0.01	Solyc10g049800.1.1	Legume lectin beta domain
−0.60	0.00	Solyc03g083910.2.1	Acid beta-fructofuranosidase
0.60	0.00	Solyc04g071610.2.1	ABSCISIC ACID STRESS RIPENING 1
0.90	0.00	Solyc11g022590.1.1	Kunitz trypsin inhibitor
Red tomatoes			
−0.90	0.00	Solyc10g049800.1.1	Legume lectin beta domain
−0.66	0.00	Solyc06g072130.2.1	Aquaporin
−0.61	0.00	Solyc03g111720.2.1	Peptide methionine sulfoxide reductase msrA
0.61	0.02	Solyc10g085480.1.1	60S ribosomal protein L24
0.89	0.00	Solyc11g022590.1.1	Kunitz trypsin inhibitor 4

^a^ Proteins that have passed the *t*-test followed by FDR correction (*p* < 0.05) when comparing protein abundance levels between Al-treated and La-treated groups. ^b^ Log2 transformed ratio from the Al-treated to the La-treated group; these datasets were used in pathway analysis using MapMan tools (Version 3.6.0RC1) [29,30]. ^c^ Accession number in the International Tomato Annotation Group (ITAG) 2.4 protein database.

**Table 2:** Significant proteins in one of the tomato ripening stages.

Cluster	Protein Accessions ^a^	Log2 Fold (Al/La) ^b^			Protein Description
		MG ^c^	Turning	Red	
Cluster 1	solyc01g060070.2.1	0.61d	0.01	0.02	Mitochondrial Tim17/22
Cluster 2	solyc01g007920.2.1	0.09	0.61d	0.84d	Isochorismatase
	solyc06g065940.2.1	0.63d	0.23	0.31	FRS6 MULE transposase
	solyc04g073990.2.1	0.89d	0.34	0.17	Annexin
	solyc11g022590.1.1	0.06	0.9d	0.89d	Kunitz trypsin inhibitor 4
	solyc04g071610.2.1	0.01	0.6d	0.4	ABA/WDS induced protein
Cluster 3	solyc06g060340.2.1	−0.7d	0.18	0.01	Photosystem II 22 kDa protein
	solyc01g007500.2.1	−0.9d	0.02	0.08	Photosystem II, PsbB
	solyc01g007330.2.1	−0.74d	0	0.01	RUBISCO, large subunit.
	solyc11g062130.1.1	−0.69d	0.01	−0.02	Mitochondrial ADP/ATP carrier proteins
	solyc06g073190.2.1	−0.63d	0.01	0.1	Carbohydrate/purine kinase, PfkB
	solyc03g122310.2.1	−0.84d	−0.09	−0.09	Aldehyde dehydrogenase
	solyc04g039850.1.1	−0.93d	0.2	0.17	ATP synthase subunit
	solyc05g051260.2.1	−1.27d	0.1	0.16	glycosyl hydrolase
	solyc06g009190.2.1	−0.66d	−0.02	0	Pectinesterase
	solyc05g051260.2.1	−1.27	0.1	0.16	Endo-1 4-beta-xylanase
	solyc01g073970.2.1	−1.03	−0.18	0.19	Histone H3
	solyc02g085840.2.1	−0.66d	−0.05	−0.01	UV excision repair protein RAD23
	solyc12g096300.1.1	−1.37d	0.08	0.24	Ribosomal protein S6
	solyc08g074240.2.1	−0.74d	0.01	0.13	Ribosomal protein S6
	solyc01g104590.2.1	−0.93d	0.01	0.09	Ribosomal protein L3
	solyc02g086240.2.1	−0.60d	−0.15	0.19	Ribosomal protein L5
	solyc06g009210.2.1	−0.88d	0.13	0.22	Ribosomal protein L19/L19e
	solyc05g054580.2.1	−0.65d	0.22	0.14	Ribosomal protein L10
	solyc06g071720.1.1	−0.81d	0	−0.09	Ribosomal protein L15
	solyc08g007620.1.1	−0.66d	0.06	0.09	Peptidase S8, subtilisin-related
	solyc07g053650.2.1	−0.7d	−0.04	−0.13	26S proteasome regulatory subunit
	solyc02g094470.2.1	−0.97d	0.24	0.26	Mitochondrial phosphate carrier protein
	solyc11g069430.1.1	−0.67d	0.24	−0.16	Aquaporin
	solyc09g092380.2.1	−0.7d	−0.1	0.01	Adenosylhomocysteinase
	solyc06g075540.2.1	−0.8d	−0.12	0.08	Phosphatidyl synthase
cluster 5	solyc09g065260.1.1	0	−0.59	−0.64d	Blue copper protein
	solyc06g072130.2.1	−0.13	−0.41	−0.66d	Aquaporin
	solyc06g034040.1.1	0.07	−0.49	−0.6d	Oleosin
cluster 7	solyc07g066610.2.1	−0.68d	0.4	0.28	Phosphoglycerate kinase
	solyc11g068540.1.1	−0.51	0.6d	0.54	N-carbamoylputrescine amidase
	solyc04g063290.2.1	−0.68d	0.6d	0.41	Ribosomal protein S5
cluster 9	solyc01g007380.1.1	−0.62d	−0.1	−0.15	Cytochrome f
	solyc12g044600.2.1	−0.63d	−0.21	−0.14	NADP-dependent malic enzyme
	solyc01g094200.2.1	−0.6d	0.06	−0.07	NAD-dependent malic enzyme
	solyc09g057650.2.1	−0.67d	−0.2	−0.23	Ribosomal protein S8e
cluster 10	solyc03g005650.1.1	0.65d	−0.07	−0.2	Cc-nbs-lrr, resistance protein

^a^ Accession number in the International Tomato Annotation Group (ITAG) 2.4 protein database. ^b^ Log2 fold changes from Al-treated to La-treated groups; these datasets were used for pathway analysis using MapMan [29,30]. ^c^ Mature green (MG) stage tomatoes. ^d^ Proteins showing significant changes (Log2, ±0.6-fold, Al/La; *p* < 0.05).

## Supplementary Materials

The Supplementary Materials are available online.
