# Supplementary material for: Effects of Al3+ and La3+ Trivalent Metal Ions on Tomato Fruit Proteomes
Source: Proteomes. 2017 Feb 11;5(1):7. doi: 10.3390/proteomes5010007 (PMC5372228; doi:10.3390/proteomes5010007)
Supplement: Supplementary file 1 [file proteomes-05-00007-s001.pdf]

Supplementary Materials: Effects of Al^{3+} and La^{3+} Trivalent Metal Ions on Tomato Fruit Proteomes

Sasikiran Sangireddy, Ikenna Okekeogbu, Zhujia Ye, Suping Zhou, Kevin J. Howe, Tara Fish and Theodore W. Thannhauser

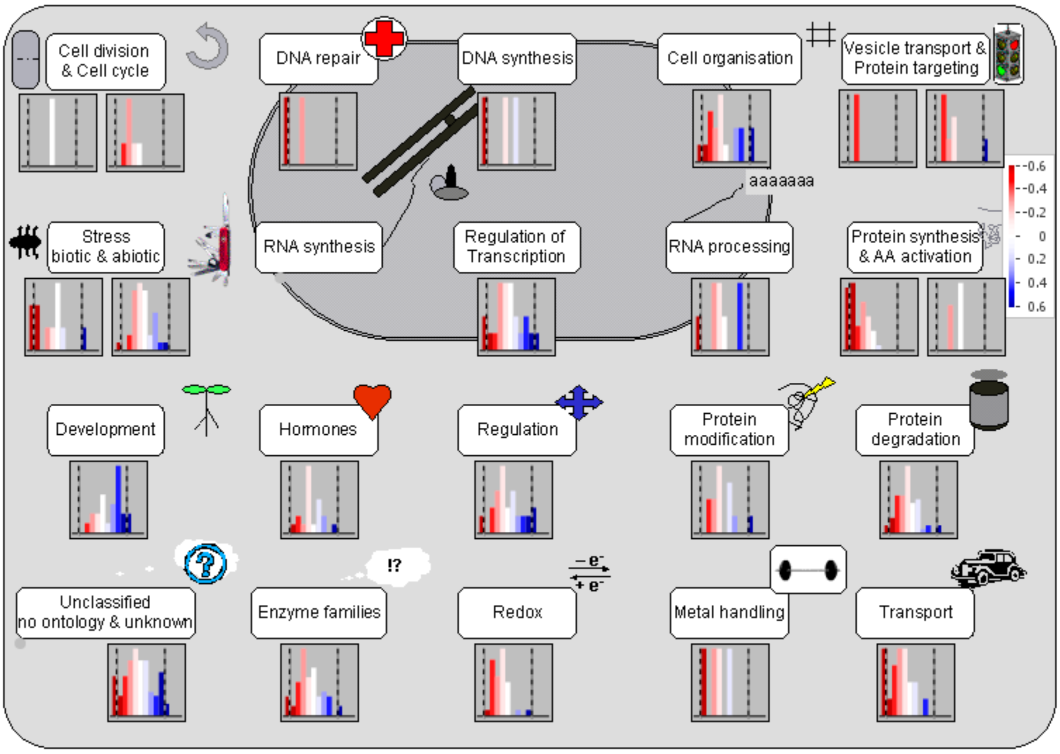

(**A**)

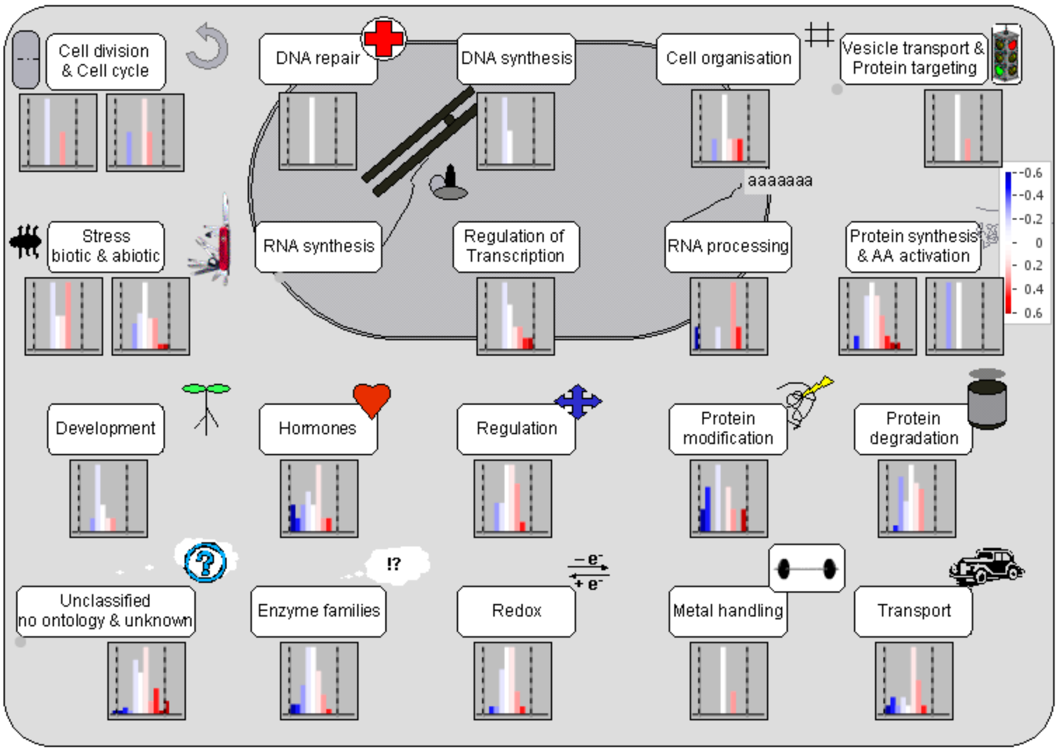

(**B**)

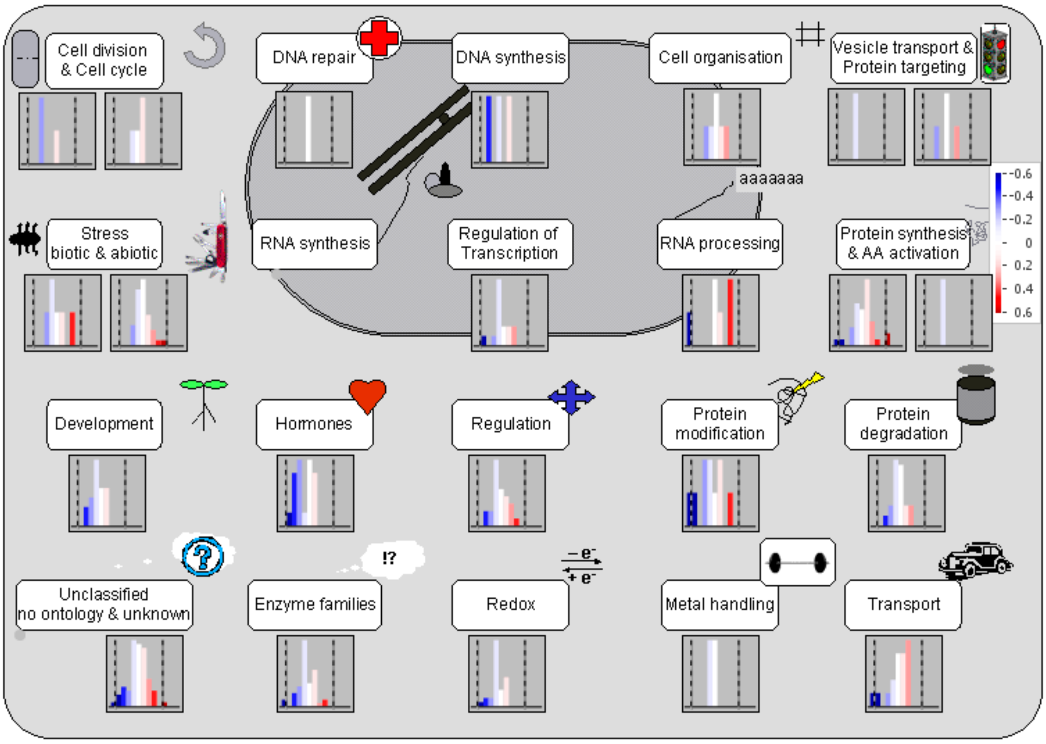

(C)

Figure S1. An overview of functional pathways in tomato fruits affected in response to Al treatments. The graph was generated using the Metabolism Overview in MapMan [29,30]. The intensity of the color change corresponds to the scale created based on the log2 fold of the respective protein from Al-treated to La-treated tomato fruit tissues. (**A**) Mature green (MG) stage tomatoes; (**B**) turning stage tomatoes; (**C**) red tomatoes.

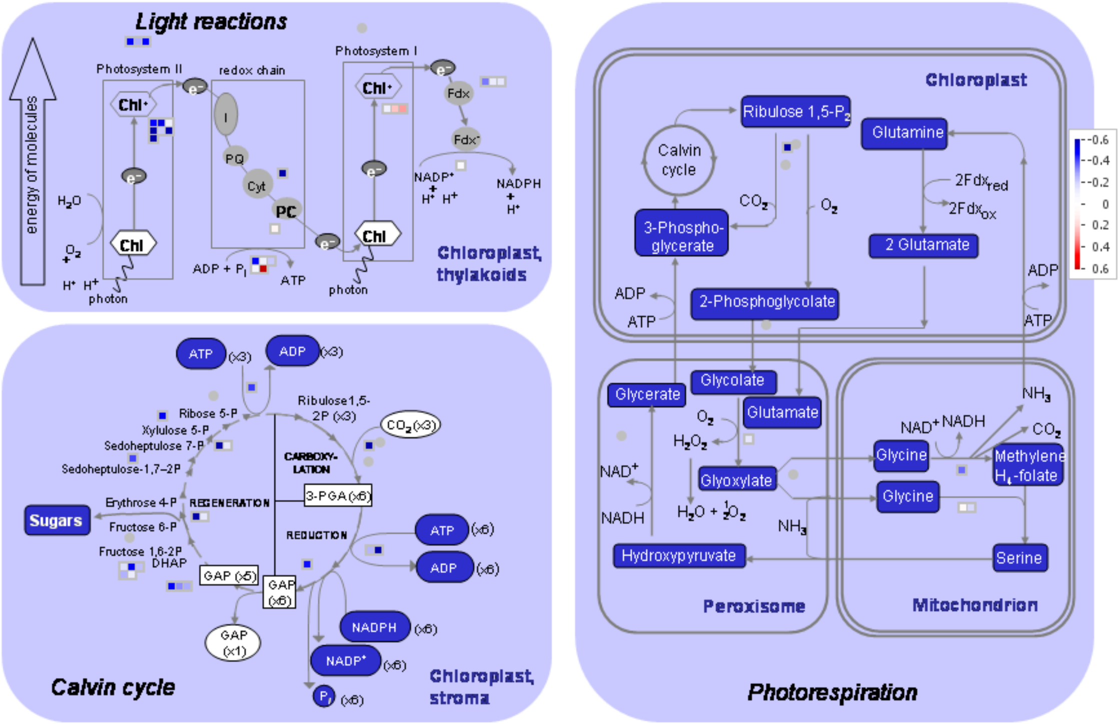

Figure S2. Photosynthesis pathways in mature green (MG) stage tomatoes in response to Al and La treatments. The graph was generated using the Metabolism Overview in MapMan [29,30]. The intensity of the color change corresponds to the scale created based on the log2 fold of the respective protein from Al-treated to La-treated tomato fruit tissues.
